# Waterpipe use in Germany (2018 – 2024): Prevalence and sociodemographic differences in age of initiation

**DOI:** 10.25646/13284

**Published:** 2025-08-27

**Authors:** Stephanie Klosterhalfen, Wolfgang Viechtbauer, Daniel Kotz

**Affiliations:** 1 Heinrich Heine University Düsseldorf, Medical Faculty and University Hospital Düsseldorf, Centre for Health and Society (chs), Addiction Research and Clinical Epidemiology Unit, Institute of General Practice (ifam), Düsseldorf, Germany; 2 Maastricht University, Mental Health and Neuroscience Research Institute, Department of Psychiatry and Neuropsychology, Maastricht, The Netherlands; 3 University College London, Department of Behavioural Science and Health, London, United Kingdom

**Keywords:** Waterpipe use, Tobacco use, Prevalence, Prevalence trends, Sociodemographic differences, Age of initiation, Face-to-face interviews

## Abstract

**Background:**

Waterpipe (WP) use poses not only a risk of nicotine dependence but also additional health hazards. This study examined trends in WP use in Germany, focusing on prevalence by age group and differences in initiation age.

**Methods:**

We analysed data from 76,239 respondents (≥ 14 years) from the German Study on Tobacco Use (DEBRA); a series of bi-monthly national surveys using face-to-face interviews at home (2018 – 2024). Prevalence trends were modelled using binomial logistic regression models with restricted cubic splines.

**Results:**

The prevalence of WP use decreased over time, to an estimated 0.9 % (95 % CI = 0.6 – 1.2) by mid-2024. This prevalence is made up of 0.1 % (95 % CI = 0.0 – 0.2) 14- to 17-year-olds, 0.3 % (95 % CI = 0.2 – 0.6) 18- to 24-year-olds, 0.3 % (95 % CI = 0.2. – 0.4) 25- to 39-year-olds, and 0.2 % (95 % CI = 0.1 – 0.3) people aged 40 years and older. WP use increased until 2020 up to 2.8 % (95 % CI = 2.3 – 3.4), remained stable for two years and then decreased, especially among people between 25 and 39 years of age. The proportion of 14- to 17-year-old users and users aged at least 40 years remained stable over the years at a low level. Median initiation age was 18 years (25th percentile: 16 years; 75th percentile: 22 years). A lower initiation age was associated with male gender and lower income.

**Conclusions:**

WP use increased from 2018 – 2020, stabilised from 2020 – 2022, and then decreased until 2024. Median initiation age was 18, with males and people with lower income starting at a younger age. Targeted public health interventions, focusing on younger males and those with lower socioeconomic status, are needed to prevent early use.

## 1. Introduction

A typical modern waterpipe (WP), also known as shisha, narghile, or hookah according to regional origin, consists of [1]: a water body, which is filled with water, an attached smoke column plus tobacco head, which is usually filled with flavoured WP tobacco (like apple, cherry, watermelon), and at least one flexible hose with a mouthpiece. By inhaling through the hose, a vacuum is created in the water body so that the smoke passes through the smoke column into the water, cools down in the meantime, and is then inhaled into the lungs [2, 3].

Due to the fruity taste and the less irritating way of smoking, the consumption of WPs can lead to a more pleasant and longer smoking experience and thus foster a higher nicotine intake [4]. Apart from the risk of nicotine dependence, WPs poses additional health risks: WP smoke has potentially harmful chemicals, including several human carcinogens and other toxic products that affect the oral and upper aero-digestive tract [5]. Thus, WP use may increase the risk of cardiovascular disease [6–8], various cancers – including oesophageal cancer, lung cancer, head and neck cancer [7, 9–12] – and chronic obstructive pulmonary disease [6, 7, 13].

Representative population surveys across various countries have collected data on WP use trends over several years [14–17]. Analysis of major surveys from 2010 – 2019, such as the Global Youth Tobacco Surveys and the US National Youth Tobacco Survey [15], revealed high WP use among 12- to 16-year-olds, especially in Europe and the Eastern Mediterranean. In half of the surveyed countries, WP prevalence increased or remained stable, while it decreased in the other half [15]. A systematic review of studies from 68 countries confirmed the high WP prevalence among youth and adults, particularly in Europe, with some countries, like the US, showing initial increases followed by decreases [14]. Similarly, EU data highlighted variability in WP use trends, with notable increases and subsequent decreases in several member states [17].

Adolescents and young adults are particularly vulnerable to starting tobacco use, which can have long-term health consequences. Focusing on different age groups is crucial, as smoking behaviours vary by age, and tailored prevention strategies are needed. Understanding trends in specific groups helps to develop more effective interventions. In Germany, there has been a decrease in the proportion of adolescents and young adults who smoke cigarettes over the last 15 years, while the use of WP has been rising in young adults [18]. The exclusive use of WPs, electronic (e)-cigarettes, or e-WPs was more widespread in 2016 than the exclusive consumption of tobacco cigarettes among German adolescents [18]. Prevalence of current WP use in 11- to 17-year-olds has not changed significantly between 2009 to 2017 (2009 – 2012 = 9.0 %, 2014 – 2017 = 8.5 %) in Germany [19]. National surveys such as the German Drug Affinity Study (Drogenaffinitätsstudie) showed a marked decrease in current WP use in the age group of 12- to 17-year-olds from 20.7 % in 2007 to 7.4 % in 2023, as well as among 18- to 25-year-olds from 44.3 % to 25.4 % [20]. The Epidemiological Survey of Substance Abuse (Epidemiologischer Suchtsurvey – ESA) has reported a recent increase in current WP use among women aged 18 to 24 years – from 13.5 % in 2018 to 14.7 % in 2021 – while usage among men in the same age group decreased from 22.4 % to 18.4 % [21]. Consistent with this, the regionally conducted SCHULBUS study (2015 – 2018) among 14- to 17-year-old students in Hamburg also showed a decrease in WP use among 14- to 15-year-olds from 22.8 % in 2009 to 11.7 % in 2018, and a more heterogenous trend among 16- to 17-year-olds, with a decrease from 30.0 % in 2009 to 20.7 % in 2015, followed by a rise back to 30.0 % in 2018 [22].

**Key messages**

► Waterpipe (WP) use in Germany has decreased in recent years, with prevalence estimated at 0.9 % by mid-2024 in the total population.
► The decline has been most pronounced among adults (25 to 39 years), while use among 14- to 17-year-olds remains stable at a low level.
► Policy measures, such as higher WP tobacco taxes and stricter packaging regulations, alongside social changes post COVID-19, including evolving social norms and changes in socialising habits, may have contributed to this trend.
► In Germany, WP initiation typically occurs at a median age of 18, later than in Middle Eastern or African countries where use is more widespread.
► Males and people with lower socioeconomic status tend to initiate using WP earlier, while factors like migration background and concurrent substance use do not significantly influence initiation age.

Nowadays, there is a wide variety of nicotine and/or tobacco-containing products to buy, so the conventional cigarette may no longer be the only common product which initiates nicotine consumption. In the US, the average age of first WP use is around 14 years, which is somewhat higher compared to the average age of cigarette initiation at 12 years [23]. In other countries, the average starting age for WP use is reported to be later, around 18 years [24]. Nevertheless, there is evidence that WP use is relevant, particularly for young adults and is increasingly becoming one of the first tobacco products used [25, 26]. It is frequently cited as an initial product in the onset of nicotine use, especially among youth who have not previously used cigarettes [27]. International studies also investigate whether the use of WPs can be seen as an ‘easy’ gateway into a future ‘smoking career’ and whether it influences the use of further tobacco and nicotine products. Recent study results support this ‘gatewayhypothesis’ for WP use [28–30].

Findings from the US Population Assessment of Tobacco and Health (PATH) Study conducted between 2013 and 2017 among young adults (ages 18 to 24) suggest that males typically initiate WP use at an earlier age than females [31]. Furthermore, the study identified disparities based on ethnic backgrounds, revealing that people with Hispanic heritage tend to initiate WP use earlier in life than those with other ethnic backgrounds. Additionally, people with prior exposure to other tobacco or nicotine products tend to initiate WP use at a younger age compared to individuals without any history of using these products. However, data concerning person characteristics and the age of WP initiation are not yet available for Germany.

Monitoring trends in WP use across different age groups is essential to understand usage patterns over time, identify distinct risk profiles, and develop targeted prevention strategies. While some national surveys in Germany have provided valuable insights [19–22, 32–34], they have been conducted only periodically and often do not include data from the most recent years [19, 22, 33]. A more detailed and up-to-date trend analysis is therefore needed to capture recent developments in WP use, particularly among adolescents, and young adults.

While prevalence data offer insight into how widespread WP use is within certain age groups, understanding age of initiation and its social determinants provides important context. Earlier initiation of WP may increase the likelihood of sustained use, which in turn increases health risks. Therefore, this study investigates both trends in WP use over time and the individual characteristics associated with initiation age.

The specific research questions were:

1. What has been the prevalence of WP usage in the population in general, and in the age groups 14 to 17, 18 to 24, 25 to 39, and ≥ 40 years specifically?
2. Does the age of initiation of WP use differ among people regarding gender, educational attainment, migration background, income, and dual use of e-cigarettes or tobacco cigarettes?

## 2. Methods

### 2.1 Study design

We used data from the German Study on Tobacco Use (DEBRA: ‘Deutsche Befragung zum Rauchverhalten’), an ongoing cross-sectional survey that collects data every other month through computer-assisted face-to-face household interviews in representative samples of approximately 2,000 people aged 14 years or older residing in Germany [35]. DEBRA is implemented as part of an omnibus survey, meaning that questions on tobacco and nicotine use are asked alongside items on other, unrelated topics. Respondents are selected by random stratified sampling (50 %) and quota sampling (50 %, details in [36]). For the present study, we used data from July 2018 (wave 13) to July 2024 (wave 49). A total of 76,239 people were interviewed during this period. The inclusion of the WP consumption question in wave 13 marks the beginning of its data collection.

The study protocol with the analysis plan was pre-registered on the Open Science Framework [37].

### 2.2 Measures

The general DEBRA survey with all questions and response options is openly accessible [38].

In this study, the term WP exclusively refers to the traditional tobacco-based WP. It does not include electronic WPs (e-shisha), which are often liquid-based and may not contain nicotine.

#### Prevalence of WP use

After a brief introductory text on the topic of WP use ‘The following questions deal with the waterpipe, also called shisha or hookah. In a waterpipe, smoke is cooled in a water bowl and inhaled through a hose. The smoke often smells and tastes fruity because it is flavoured’. We asked: ‘Have you ever used a waterpipe?’. Response options were: ‘(1) Yes, I have used them until today [defines current WP users], (2) Yes, I used them regularly in the past, but don’t use them anymore, (3) Yes, I tried them earlier, but don’t use them anymore, (4) No, I have never used them, (5) No response’.

#### Age of initiation of WP use

Current WP users were asked: ‘In which year or at what age have you begun using waterpipes? Please give only a single answer: please give either the year or the age’. Response options were: ‘(1) Year: ____, (2) Age: ____, (3) Don’t know, (4) No response’. If a year was given, the age of initiation was calculated by subtracting the years since the first use from their current age. For example, if someone initiated in 2020 and is 20 years old in 2024, the age of initiation would be 16. Both inputs are then combined into a single variable for age of initiation of WP use.

#### Concurrent use of WPs and e-cigarettes and/or tobacco cigarettes

Concurrent users of WPs were defined as all respondents who were current WP users (see above) as well as current cigarette smokers (defined as people who (1) ‘(…) smoke cigarettes every day’, (2) ‘(…) smoke cigarettes, but not every day’) and/or current e-cigarette user (defined as people who stated (1) ‘Yes, I have used them until today’).

#### Demographic characteristics

In order to reflect distinct life stages and consumption patterns, especially given that WP use is concentrated among younger people, we stratified age into four groups: 14 to 17, 18 to 24, 25 to 39, 40 years and older, following age categorisation that is also commonly used in nationwide studies [21]. This allowed for a more nuanced understanding of prevalence trends across age. Gender was self-reported as female or male (22 respondents with diverse gender were excluded from the analyses). Migration background was assessed in a binary format (migration background: yes/no). A migration background was defined as present if at least one parent of the respondent was not born in Germany. This operationalisation follows pragmatic data collection standards, as also applied in official statistics in Germany, such as the Microcensus. German equivalents to education attainment listed from lowest to highest: low = no qualification/junior high school equivalent, middle = secondary school equivalent, high = advanced technical college equivalent/high school equivalent. Net monthly household income: low = approximately < 20th income percentile, middle = approximately 20th to 80th income percentiles, and high = approximately > 80th income percentile. These categories reflect the distribution of income in Germany [39–41]. The calculation was based on an equalisation technique provided by the Organisation for Economic Cooperation and Development (OECD-modified equivalence scale) [42], adjusting total net household income (after tax and other deductions) for household size and composition.

#### Concurrent nicotine and tobacco product use

Concurrent use is defined as the use of e-cigarettes and/or tobacco cigarettes in addition to WP.

### 2.3 Statistical analyses

To address research question 1 (prevalence of WP use), we plotted prevalences (in %) over time for the total number of 76,239 (n_weighted_ = 76,233) respondents, along with pointwise 95 % confidence intervals (CI) using the Clopper-Pearson method. This was done both for the total group of current WP user (n_weighted_ = 1,742) and stratified by age groups (14 to 17, n_weighted_ = 133; 18 to 24, n_weighted_ = 747; 25 to 39, n_weighted_ = 632; ≥ 40 years, n_weighted_ = 230) using weighted data. Weighting aimed to approximate the sample to the German population in terms of age, gender, household size, level of education and region (details in [43]).

To model trends, we used binomial logistic regression models utilising restricted cubic spline terms for wave with knot positions at January 2020, July 2021, and January 2023. Splines allow modelling smooth, flexible curves over time instead of assuming a strictly linear trend – capturing gradual increases or decreases more accurately. To account for possible overdispersion, a random effect for wave was added to the model. These knots were chosen, to be approximately evenly spaced across the study period (July 2018 to July 2024), allowing the model to flexibly capture potential non-linear trends in prevalence over time while avoiding overfitting.

Model fit was compared across a spline model, a linear trend model, which in turn was compared with a model of no trend via likelihood ratio tests. In addition to likelihood ratio tests, model comparison criteria such as the Akaike Information Criterion (AIC) and the Bayesian Information Criterion (BIC) were evaluated.

Each spline model used five parameters (including the intercept). Even in the smallest subgroup (14 to 17 years), the number of observed cases is sufficient based on commonly cited rules of thumb for logistic regression. From a power perspective, these models are adequately supported by the sample size, and the relatively narrow confidence intervals around trend lines confirm the robustness of the estimates.

To address research question 2 (differences in the age of initiation of WP use), we conducted a descriptive analysis of the medians for initiation age, including interquartile range, aggregated across all waves (13 – 49). Differences were tested using the Mann-Whitney U test for two independent groups (e.g., gender: female vs. male; migration background: yes vs. no migration background; concurrent use: concurrent user vs. non-concurrent user) and the Kruskal-Wallis test for comparisons among three or more independent groups (e.g., educational attainment: low, middle, high; income: low, middle, high). While the overall test indicated a significant association between income level and age of initiation, the Bonferroni correction was applied post hoc to control for potential inflation of Type I error due to multiple pairwise comparisons. This adjustment, although not pre-specified in the study protocol, was deemed necessary for a more rigorous interpretation of the subgroup differences.

The analyses were conducted using R (version 4.4.1) and IBM SPSS Statistics (version 29.0.0.0).

## 3. Results

Of the 76,239 respondents interviewed from 2018 to 2024, 1,491 (2.0 %) reported WP use. We excluded 256 (0.3 %) from the analyses who did not provide a response regarding WP use. A total of 969 respondents (65.0 % out of 1,491 WP users) used WP and in addition e-cigarettes and/or tobacco cigarettes (concurrent use).

In the sample of current WP users, 44.5 % (95 % CI = 42.0 – 47.1) were younger than 25 years. The majority of current WP users was male (63.2 %, 95 % CI = 60.7 – 65.7), and 32.7 % (95 % CI = 30.3 – 35.1) reported having a migration background. Additionally, 67.7 % (95 % CI = 65.3 – 70.1) of them concurrently smoked tobacco cigarettes, pipes, or cigars (Table 1).

###  

#### Prevalence of WP use

**Figure 1:**
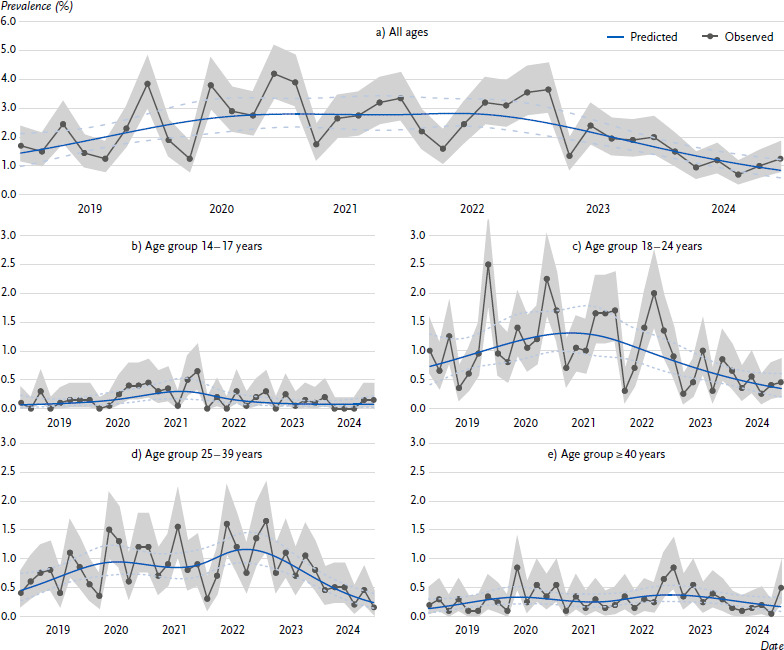
Trend in prevalence of waterpipe use in the German population between 2018 – 2024 ^*^. Source: DEBRA study 2018 – 2024 ^*^The graphs show the prevalence (%) over time (2018 – 2024) for all ages and stratified by different age groups: (a) all ages, (b) for 14- to 17-year-olds, (c) 18- to 24-year-olds, (d) 25- to 39-year-olds, and (e) for people ≥ 40 years. Each plot includes data points from each wave with 95 % confidence intervals and a smoothed trend line (blue).

Figure 1^1^ shows the modelled trends in the prevalence of WP use since 2018. The spline model fits significantly better compared to the linear and no trend models (see Annex Table 1 for the results of the model comparisons). Results from the spline regression models are provided in Annex Table 2. From our perspective, the models capture the overall trend in observed prevalence’s well, suggesting that the model specification is appropriate and stable. In the total population, the prevalence had increased to 2.8 % (95 % CI = 2.2 – 3.4) by spring 2020 and then remained stable at this level for approximately two years. The prevalence consistently decreased from spring 2022, reaching a low of 0.9 % (95 % CI = 0.6 – 1.2) by summer 2024 (Figure 1a). The proportion of 14- to 17-year-old users in the total population showed an increase, rising from 0.1 % (95 % CI = 0.1 – 0.2) at the end of 2019 to 0.3 % in mid-2021 (95 % CI = 0.2 – 0.5). Following this increase, the prevalence levelled off and returned to the initial level of 0.1 % (95 % CI = 0.0 – 0.2) by mid-2024 (Figure 1b). For 18- to 24-year-olds, WP use increased until mid-2021, peaking at 1.3 % (95 % CI = 1.0 – 1.8). Thereafter, it decreased continuously, falling to a low of 0.3 % (95 % CI = 0.2 – 0.6) (Figure 1c). The overall prevalence of WP use for people aged between 25 and 39 years, exhibited an almost linear increase from 0.5 % (95 % CI = 0.3 – 0.8) in mid-2018 to 0.9 % (95 % CI = 0.7 – 1.1) in 2020, remained stable until mid-2021, and another estimated peak at 1.2 % (95 % CI = 0.9 – 1.4) in the second half of 2022. Subsequently, the prevalence decreased almost linearly, reaching a low of 0.2 % (95 % CI = 0.1 – 0.3) at the last measurement point in mid-2024 (Figure 1d). For people aged at least 40 years, the prevalence slightly increased from 0.1 % (95 % CI = 0.1 – 0.3) in mid-2018 to 0.3 % (95 % CI = 0.2 – 0.5) in 2020. This was followed by a relatively stable period, and since the second half of 2022, there has been a steady slight decrease, reaching the most recent value of 0.2 % (95 % CI = 0.1 – 0.3) in mid-2024 (Figure 1e).

#### Group differences in the age of initiation of WP use

The overall median age of initiation, calculated across all respondents and survey waves, was 18 years (25th percentile: 16 years, 75th percentile: 22 years). Percentile values stratified by calendar year are provided in Annex Table 3.

Table 2 presents group differences in the age of initiation of WP use based on gender, migration background, concurrent use, educational attainment and net monthly household income. A significant difference was observed between females and males, with males tending to initiate WP use at a younger age than females. The 25th, 50th (median) and 75th percentiles for males were 16, 18, and 21 years, respectively, while for females, they were 16, 18, and 25 years.

Income level was also significantly associated with the age of initiation. Pairwise comparisons revealed a significant difference between low- and high-income groups (adjusted *p* = 0.012), while no significant differences were found between other pairs after applying the Bonferroni correction. No significant differences were found for migration background, concurrent use of other nicotine products, or educational attainment.

## 4. Discussion

Our results indicate that the prevalence of WP use in Germany has decreased since 2018 to an estimated 0.9 % by mid-2024, with a particularly noticeable decrease beginning in early 2022. This trend may indicate a decrease in WP popularity, particularly among people aged 25 to 39 years. Together with further national data a more nuanced picture of WP consumption can be drawn. The German Drug Affinity Study reported a marked decline in WP use among adolescents (12 to 17 years) from 20.7 % in 2007 to 7.4 % in 2023, among young adults (18 to 25 years) from 44.3 % to 25.4 % [20]. In contrast, ESA found an increase in current WP use among women aged 18 to 24 years between 2018 and 2021, while use among men in this age group declined [44]. Beyond quantitative data, qualitative studies such as the Shisha-M Study offer insight into usage motives [34]. These findings provide important context for interpreting the DEBRA study results. They underscore the value of integrating national quantitative and qualitative data sources to build a comprehensive understanding of WP use patterns and inform prevention strategies in Germany.

Internationally, similar trends have been observed, although the prevalence of WP use varies significantly across countries. Additionally, our findings suggest that males tend to initiate WP use at an earlier age than females.

The strengths of our study include the use of nationally representative samples, along with six years of bi-monthly data collection, providing a robust data set for analysing trends over time. The comprehensive data collection across various age groups and socioeconomic and demographic categories allows for a more nuanced analysis of WP use patterns in Germany. Moreover, the implementation of face-to-face interviews helped minimise missing data, ensuring higher data accuracy and reliability. However, there are limitations. The relatively low prevalence of WP use in Germany limited the sample size and statistical power, especially for subgroup analyses. Although some seasonal variation might be visually suspected in Figure 1, this was not explicitly modelled. Incorporating seasonality would considerably increase model complexity and was therefore not pursued. Nonetheless, the random effect for wave may absorb some of the unexplained variation, including possible seasonal effects, and helps to account for between-wave heterogeneity. The spline model generally showed better fit (i.e., lower AIC and BIC values), although in the 14 to 17 age group the BIC favoured simpler models. These findings suggest that in this group, minor fluctuations – such as a small increase around 2021 – should be interpreted cautiously. Additionally, social desirability bias may have led to underreporting of WP use, particularly among younger participants, given the legal restrictions on smoking WP for people under 18. Moreover, the question on WP use did not include a specific time frame – like usage in the last 30 days – limiting comparability with other surveys. Furthermore, as the survey was only available to German speakers, individuals with a migration background may be underrepresented, even though previous research indicates that people with a migration background are more likely to smoke WP. Lastly, the aggregated analysis from 2018 to 2024 provides a general overview, but participant characteristics and their associations with WP use may have evolved over this period, potentially limiting the applicability of the findings to specific time points.

Internationally, similar trends are evident, with the prevalence of current WP smoking showing significant variation across countries. In some, including Germany, there has been a notable decrease in WP use over recent years [14,15]. For example, data from the 2021 National Health Interview Survey in the United States also reported a WP prevalence of 0.9 % among adults, with higher rates among those aged 18 to 24 years (1.8 %) and 25 to 44 years (1.6 %) [45]. Additionally, findings from the Population Assessment of Tobacco and Health (PATH) Study observed a steady decrease in current WP use from 2013 to 2021, with prevalence among adolescents decreasing from about 1.7 % to 0.05 % and among adults from about 2.2 % to 1.2 % [46].

Since 2022, a decrease in WP use has been observed in Germany, likely influenced by several regulatory and social factors. Recent policy changes may have played a role: According to Section 2 of the Tobacco Tax Act (TabStG), starting January 1, 2022, an additional tax on WP tobacco was introduced, initially set at 15 € per kilogram and scheduled to increase annually, reaching 23 € per kilogram by 2026 [47]. This rising cost may discourage WP consumption, particularly among people with limited disposable income. Additionally, new packaging regulations implemented from July 1, 2022 to July 1, 2024, by the Ordinance on the Amendment of the Tobacco Tax Regulation (TabStÄndV) restrict manufacturers to producing only 25-gram packages, potentially reducing WP use by limiting bulk purchasing options and encouraging smaller, more occasional purchases [48]. While these data do not allow us to conclusively link policy changes to the decrease in WP use, the timing suggests these measures could be influencing consumption patterns. Furthermore, the COVID-19 pandemic likely impacted WP use. WP smoking is typically a group activity, and the pandemic’s disruption of social routines may have led to longer-term shifts in WP use habits. This combination of regulatory and social factors could be driving the decreasing trend observed in WP prevalence since mid-2021. Additionally, the decrease in WP use could, at least in part, be influenced by the concurrent increase in e-cigarette consumption in Germany since mid-2021 [49], potentially reflecting a shift in product preferences.

The data from our study indicate that WP users began their consumption at a median age of 18.0 years. A German qualitative study reported that initiation typically occurs between the ages of 14 and 18 years [34]. In contrast, studies of adolescents from several African countries, and US middle and high school students, report a median initiation age of 14.1 years (interquartile range (IQR) = 12.4 – 15.3) [23, 50], while data from Sarajevo, Bosnia-Herzegovina, show initiation ages between 15 and 16 years [51]. This suggests that German WP users tend to start at a later age. Similarly, people in Turkey begin WP use relatively late, with an average initiation age of 17.7 years [52]. A systematic review further highlights that the initiation age of WP use varies across countries, typically ranging from 14 to 18 years [53]. This higher age of initiation in Germany might reflect broader trends, as Ebrahimi et al. reported a growing prevalence of WP smoking among adults over time, suggesting that people may begin smoking later in life [46]. The authors attribute this to the fact that adolescents often lack access to WP lounges and may find the equipment and process more difficult to manage, as it requires a certain level of experience and independence typically associated with older age groups. Additionally, greater autonomy from parental supervision and stronger integration into social circles that promote WP smoking likely contribute to the higher prevalence observed in young adults.

Our findings also show a significant gender difference in the age of initiation, with males reporting an earlier start compared to females. Similar results were reported by Sharapova et al. using data from the National Youth Tobacco Surveys (2014 – 2016) [23], which found that males tended to start smoking WPs at a younger age.

Age differences in WP use are an important aspect of understanding smoking initiation and patterns of use. Marcon et al. reported an increase in smoking initiation among youths aged 15 years or younger across 17 European countries, although their data primarily pertained to cigarette smoking [55]. We found no significant difference in the age of initiation between concurrent users and exclusive WP users. However, the literature presents mixed results. A study conducted in Iran found that dual users (WPs and cigarettes) were on average older than exclusive WP users [55]. Conversely, a study from Pakistan showed that dual users were younger than those who only used WP [56].

Policymakers and clinicians may consider these factors when addressing tobacco control strategies and preventive health measures, particularly focusing on younger adults and people with limited financial means. Regulatory measures, such as increased taxation and packaging restrictions, appear to influence WP consumption, potentially reducing its appeal to cost-sensitive groups. Furthermore, social factors such as the COVID-19 pandemic and the rise of e-cigarettes may continue to shape WP use patterns in Germany.

While this study provides valuable insights into the trends of WP use in Germany, several questions remain unanswered. Future research should explore the long-term effects of the regulatory changes introduced in 2022 and their sustained impact on WP consumption. Additionally, further studies are needed to better understand the mechanisms behind the shift towards e-cigarettes and whether they are serving as a substitute for WP use. Understanding the socioeconomic and cultural factors that influence WP initiation, especially in younger populations, will also be crucial in shaping future interventions. Finally, the role of social networks in promoting or deterring WP use warrants further exploration.

## Figures and Tables

**Table 1:** Sample characteristics (unweighted)^*^. Source: DEBRA study 2018 – 2024

	Total % (absolute numbers) n = 76,239	Current WP users % (absolute numbers) n = 1,491
**Age (years)**		
14 to 17	2.4 (1,856)	6.3 (94)
18 to 24	8.2 (6,276)	38.2 (570)
25 to 39	20.5 (15,596)	40.2 (600)
≥ 40	68.9 (52,511)	15.2 (227)
**Gender**		
Female	51.8 (39,472)	36.8 (548)
Male	48.2 (36,745)	63.2 (943)
**Migration background**		
Yes	14.1 (10,756)	32.7 (487)
No	80.0 (60,991)	57.1 (851)
**Educational attainment**		
Low	29.6 (22,571)	22.6 (337)
Middle	36.6 (27,928)	33.9 (505)
High	30.3 (23,137)	32.8 (489)
**Net monthly household income (€)**		
Low	13.2 (10,068)	22.7 (339)
Middle	63.4 (48,307)	56.9 (848)
High	23.3 (17,731)	19.9 (296)
**Smoking status**		
Current smoker	32.2 (24,536)	67.7 (1,010)
Ex-smoker	16.8 (12,790)	6.2 (93)
Never smoker	50.5 (38,520)	25.6 (381)
**E-cigarette status**		
Current user	1.6 (1,213)	11.7 (175)
Ex-user	10.1 (7,717)	32.7 (488)
Never user	87.9 (67,024)	55.5 (827)

WP = waterpipe, 95 % CI = 95 % confidence interval

^*^Data are presented as percentages (absolute numbers), aggregated for waves 13 – 49 (07/2018 – 07/2024). Data are presented as 100 % within the columns. Differences when calculating the percentages in the columns can be explained by missing data on the respective variable.

**Table 2:** Group differences in the age of initiation of waterpipe use. Source: DEBRA study 2018 – 2024

Variable	Test Statistic	*p*-value
Gender	U^1^ = 224060	0.013
Migration background	U = 159626	0.168
Concurrent use	U = 228754	0.115
Educational attainment	H^2^ = 1.278	0.528
Net monthly household income	H = 8.647	0.013

^1^ U = Mann-Whitney-U-Test

^2^ H = Kruskal-Wallis-Test

**Annex Table 1:** *p*-values for model comparisons. Source: DEBRA study 2018 – 2024

	*p*-value of linear trend versus no trend	*p*-value of non-linear trend versus linear trend
**All ages combined and stratified by age**		
Any age	0.000	0.000
14 – 17 years	0.036	0.020
18 – 24 years	0.001	0.008
25 – 39 years	0.000	0.000
≥ 40 years	0.200	0.121

**Annex Table 2:** Results from binomial logistic regression with restricted cubic splines for wave^*^. Source: DEBRA study 2018 – 2024

Age	Predictor	Coefficient (ß)	Standard Error	*p*-value
Any age	Intercept	- 4.218	0.198	< 0.001
	Spline term 1	0.626	0.237	0.008
	Spline term 2	0.456	0.212	0.032
	Spline term 3	0.727	0.526	0.167
	Spline term 4	- 0.903	0.220	< 0.001
	Random effect SD (wave)		0.263	
14 – 17 years	Intercept	- 7.248	0.613	< 0.001
	Spline term 1	1.853	0.671	0.006
	Spline term 2	- 0.138	0.613	0.823
	Spline term 3	0.575	1.574	0.715
	Spline term 4	- 0.158	0.628	0.801
	Random effect SD (wave)		0.605	
18 – 24 years	Intercept	- 4.919	0.297	< 0.001
	Spline term 1	0.654	0.359	0.068
	Spline term 2	- 0.049	0.331	0.882
	Spline term 3	0.138	0.790	0.861
	Spline term 4	- 1.024	0.341	0.003
	Random effect SD (wave)		0.402	
25 – 39 years	Intercept	- 5.384	0.255	< 0.001
	Spline term 1	0.434	0.295	0.142
	Spline term 2	0.943	0.259	< 0.001
	Spline term 3	1.154	0.666	0.083
	Spline term 4	- 1.107	0.293	< 0.001
	Random effect SD (wave)		0.268	
≥ 40 years	Intercept	- 6.621	0.415	< 0.001
	Spline term 1	0.364	0.483	0.452
	Spline term 2	0.858	0.399	0.031
	Spline term 3	1.918	1.077	0.075
	Spline term 4	- 0.415	0.413	0.315
	Random effect SD (wave)		0.404	

SD = standard deviation

^*^Spline terms represent the restricted cubic spline transformation of the wave variable (with knots at January 2020, July 2021, January 2023).

**Annex Table 3:** Median age at initiation by year. Source: DEBRA Study 2018 – 2024

Year	25th percentile	50th median	75th percentile
2018	16	18	26
2019	17	18	20
2020	16	18	20
2021	16	18	22
2022	16	18	23
2023	16	18	25
2024	15	18	20

## Footnotes

^1^ Note on how to read the figure: The value e.g., of 0.9 % refers to the proportion of the total population reporting WP use by mid-2024. In a next step, this overall prevalence was broken down by age group. This total is composed of 0.1 % among 14- to 17-year-olds, 0.3 % among 18- to 24-year-olds, 0.3 % among people aged 25 to 39 years and 0.2 % among people aged 40 years and older. The denominator (total population) is the same across all five panels.
